# Supplementary material for: SARS-CoV-2 RBD Conjugated to Polyglucin, Spermidine, and dsRNA Elicits a Strong Immune Response in Mice
Source: Vaccines (Basel). 2023 Apr 6;11(4):808. doi: 10.3390/vaccines11040808 (PMC10146165; doi:10.3390/vaccines11040808)

Figure S1. Representative plots of ICS staining and Flow cytometry analysis of RBD-specific IFN-γ-producing CD4+ and CD8+ mouse T cells

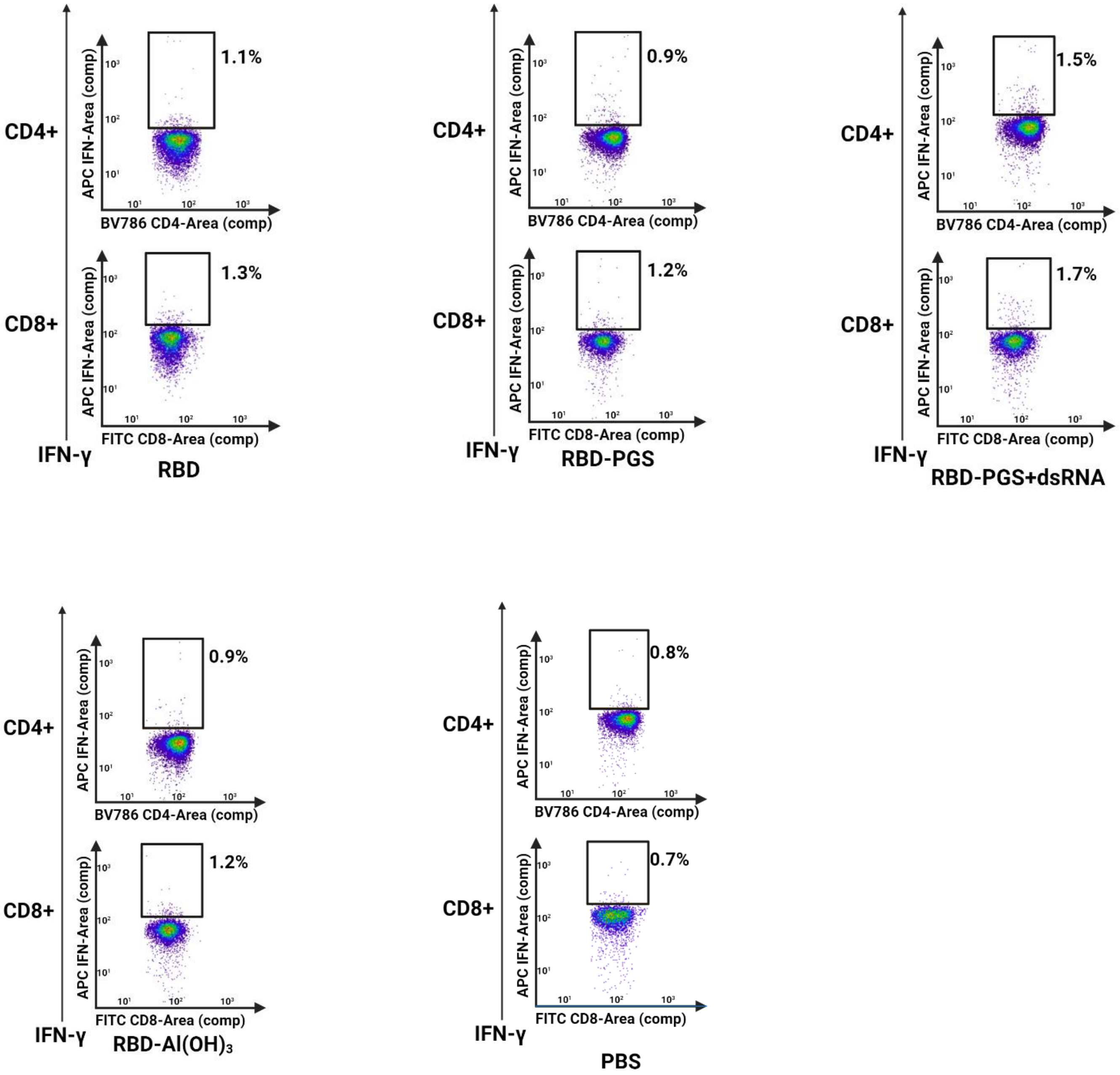

Supplement: Supplementary file 1 [file vaccines-11-00808-s001.zip › vaccines-2293845-supplementary.pdf]
